# Retinal Microvascular Changes in Association with Endothelial Glycocalyx Damage and Arterial Stiffness in Patients with Retinal Vein Occlusion: A Cross-Sectional Study

**DOI:** 10.3390/biomedicines12112564

**Published:** 2024-11-09

**Authors:** Konstantinos Pappelis, Alexia Risi-Koziona, Chrysa Agapitou, Emmanouil Korakas, John Thymis, George Pavlidis, Stamatios Lampsas, Aikaterini Kountouri, Loukia Pliouta, Ilias Georgalas, Panagiotis Theodossiadis, Vaia Lambadiari, Ignatios Ikonomidis, Irini Chatziralli

**Affiliations:** 1 2nd Department of Ophthalmology, Attikon Hospital, Medical School, National and Kapodistrian University of Athens, 12462 Athens, Greece; 2 2nd Department of Internal Medicine, Research Unit and Diabetes Centre, Attikon Hospital, Medical School, National and Kapodistrian University of Athens, 12462 Athens, Greece; 3 2nd Department of Cardiology, Attikon Hospital, Medical School, National and Kapodistrian University of Athens, 12462 Athens, Greece; 4 1st Department of Ophthalmology, G. Gennimatas General Hospital, Medical School, National and Kapodistrian University of Athens, 12462 Athens, Greece

**Keywords:** arterial stiffness, endothelial dysfunction, retinal vein occlusion, OCT-A, cardiovascular

## Abstract

Background/Objectives: To investigate the potential association between the endothelial dysfunction and arterial stiffness with retinal changes observed through optical coherence tomography (OCT) and OCT-angiography (OCT-A) in patients with retinal vein occlusion (RVO). Methods: Participants in this cross-sectional study were 28 patients with RVO. The demographic and clinical characteristics of all participants were recorded. Comprehensive ophthalmologic examinations were performed, including fundus photography, OCT and OCT-A. Endothelial dysfunction was assessed by measuring the endothelial glycocalyx thickness via the perfused boundary region (PBR5-25). Arterial stiffness was evaluated by measuring the carotid-femoral pulse wave velocity (PWV), the central systolic and diastolic blood pressures (cSBP and cDBP) and the augmentation index (Aix). For each ophthalmological outcome, we generated a saturated linear regression model with demographic and systemic vascular parameters serving as independent variables. Regression coefficients with the corresponding 95% confidence intervals (CIs) were reported. A *p* value < 0.05 was considered as statistically significant. Results: A 1 m/s increase in PWV was associated with a 0.6% reduction in inferior macular vessel density (VD) (*p* = 0.050). A 10 mmHg increase in cSBP was associated with a 0.03 mm^2^ increase in foveal avascular zone (FAZ) area (*p* = 0.033). A 1% increase in Aix was associated with a 0.005 mm^2^ increase in FAZ area (*p* = 0.008). A 1 μm increase in PBR5-25 was associated, on average, with a 4.4% decrease in superior peripapillary VD (*p* = 0.027). Conclusions: In patients with RVO, structural and microvascular retinal parameters were significantly associated with markers of endothelial dysfunction and arterial stiffness.

## 1. Introduction

Retinal vein occlusion (RVO), either central (CRVO) or branch (BRVO), is the second most common retinal vascular disorder after diabetic retinopathy, characterized by the blockage of a vein carrying blood away from the retina, and can lead to visual loss, especially due to its complications, namely macular edema and retinal ischemia [1,2]. The estimated global prevalence of RVO is approximately 0.52%, affecting about 16 million people worldwide [3]. The incidence of RVO increases with age, while there is no gender predilection [4,5]. The exact pathogenesis of RVO is poorly understood, but the main cause is thought to be the compression of the retinal vein by an atherosclerotic retinal artery, either at arteriovenous crossing sites (BRVO) or at lamina cribrosa (CRVO), where the artery and the vein share a common adventitial sheath [6]. Of note, the well-known Virchow’s triad, including hemodynamic changes (venous statis), degenerative changes of the vessel wall and blood hypercoagulability, has been involved in the pathogenesis of RVO [6].

Various cardiovascular, thrombophilic, systemic and ocular conditions predispose to RVOs. The most common risk factors for RVO development are advanced age, hypertension, atherosclerosis, hyperlipidaemia, diabetes mellitus, smoking, elevated body mass index, hyperhomocysteinemia, hypercoagulability, open-angle glaucoma and ocular inflammatory diseases [7,8]. Moreover, local inflammation may also play a role in the pathophysiology of RVO, since elevated levels of pro-inflammatory mediators, such as interleukin-6 (IL-6), interleukin-8 (IL-8), endothelin-1 and vascular endothelial growth factor (VEGF) have been found in the vitreous fluid of RVO patients [9,10,11].

It is worth noting that patients with RVO present an elevated risk for cardiovascular diseases and mortality [12,13]. Endothelial dysfunction and arterial stiffness are critical contributors to cardiovascular pathophysiology and also appear to be involved in RVO pathogenesis [14,15,16,17,18,19]. Previous studies have shown that arterial stiffness is increased in patients with BRVO independently of the blood pressure [15,17], while the augmentation index (Aix) and pulse-wave-velocity (PWV) have been found to be increased in patients with CRVO compared to controls [19]. Additionally, patients with RVO have been shown to exhibit microvascular changes on optical coherence tomography-angiography (OCT-A), including enlargement of the foveal avascular zone (FAZ), increased parafoveal capillary non-perfusion and reduced parafoveal vessel density [20,21]. However, no studies have explored the relationship between endothelial glycocalyx integrity and arterial stiffness in association with OCT-A findings in patients with RVO.

In light of the above, the aim of this study was to assess the potential association between endothelial damage and arterial stiffness with retinal changes observed on OCT and OCT-A in patients with RVO.

## 2. Materials and Methods

Participants in this cross-sectional study were patients with RVO diagnosed at the 2nd Department of Ophthalmology, National and Kapodistrian University of Athens, Athens, Greece, between January 2024 and May 2024. Diagnosis of RVO was clinically established, based on the presence of retinal hemorrhages, dilated and tortuous veins and flame-shaped or dot-blot hemorrhages, and it was confirmed though retinal imaging. Patients with other retinal diseases than RVO, corneal disease, dense cataract, uveitis, uncontrolled glaucoma with intraocular pressure (IOP) ≥ 30 mmHg, trauma and any previous intraocular surgery during the last 6 months, as well as patients with haematologic, cardiovascular, rheumatologic, infectious pathologies and malignancies, were excluded from the study. The study was in adherence with the tenets of the 1964 Helsinki Declaration and was approved by the Institutional Review Board of Attikon University Hospital (Reference number: 323/2024, 17 January 2024). Written informed consent was obtained by all participants before enrollment in the study.

The demographic characteristics and clinical data of the participants were documented. A comprehensive ophthalmologic examination was conducted, including measurement of best-corrected visual acuity (BCVA) using Snellen charts, slit-lamp examination, dilated fundoscopy, color fundus photography, swept source-OCT (SS-OCT) and OCT-A, using Topcon DRI OCT Triton Plus (Topcon, Tokyo, Japan). The machine employs an angiography ratio analysis (OCTARA) algorithm and has an A-scan rate of 100,000 A-scans per second, operating with a long wavelength scanning light of 1050 nm. It features an axial resolution of 8 µm, a lateral resolution of 20 µm and an imaging depth of 2.6 mm [22,23].

For the SS-OCT protocol, scans were performed to measure the macular area and peripapillary retinal nerve fiber layer (RNFL). The macular area was depicted using the 3D 7 × 7 mm (H) protocol, which provided measurements of the nine macular regions defined by the Early Treatment Diabetic Retinopathy Study (ETDRS) grid. This includes a central 1 mm circle representing the fovea, along with inner and outer rings measuring 3 mm and 6 mm diameter, respectively, to assess full retinal thickness. The peripapillary area was measured using the 3D Disc 6 × 6 mm protocol, which analyzed RNFL thickness across four quadrants (superior, nasal, inferior and temporal), as well as the average RNFL thickness.

**Figure 1:**
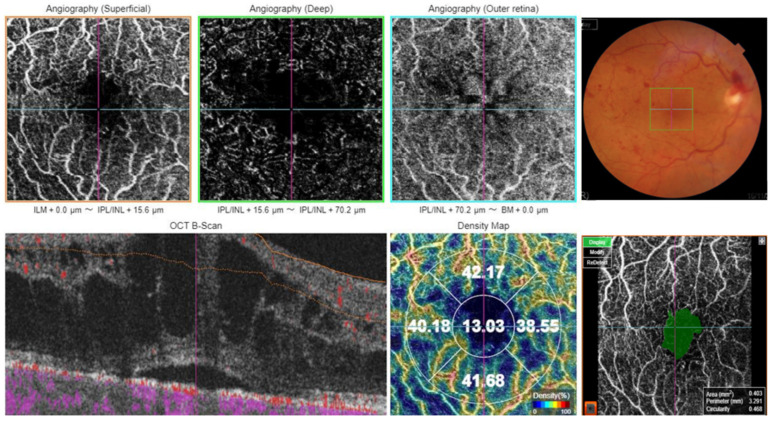
Optical coherence tomography angiography in the superficial capillary plexus, in the deep capillary plexus and in the outer retina, and color fundus photo (upper panel from left to right); optical coherence tomography, vessel density map and foveal avascular zone metrics (lower panel from left to right) in a 68-year-old female patient with central retinal vein occlusion.

For OCT-A acquisition, the 3 × 3 mm protocol was used for macular imaging. The scans were checked for foveal centration and if any decentration occurred, the ETDRS grid was manually centered on the fovea. The automated software segmented the superficial capillary plexus and macular vascular density (MVD) was measured in five regions: central, inner superior, inner nasal, inner inferior, and inner temporal subfields of ETDRS grid. Additionally, FAZ metrics including FAZ area, FAZ perimeter, and FAZ circularity index were evaluated. Figure 1 shows the density map and FAZ metrics of a study eye using this protocol. Optic nerve head angiography was performed using the 4.5 × 4.5 mm protocol, with the scan centered on the optic disc. All images were captured by the same investigator (IC). The OCT-A images were reviewed for artifacts and any images with artifacts were captured again. Only images with a scan quality score greater than 55 were included in the analysis; and poor-quality images were excluded and rescanned until acceptable quality was achieved.

In addition to ocular examinations and retinal imaging, all patients underwent evaluation of the endothelial function, by measuring the endothelial glycocalyx thickness. We assessed the integrity of the endothelial glycocalyx by measuring the perfused boundary region (PBR), which represents the area of the endothelial glycocalyx that can be penetrated by erythrocytes. The PBR is the cell-poor layer created by the separation between the red blood cell column and the plasma along the vascular lumen surface. An increased PBR value indicates deeper blood cell penetration into the glycocalyx, serving as an accurate marker of reduced glycocalyx thickness and endothelial dysfunction [24,25,26]. Therefore, PBR is inversely proportional to endothelial glycocalyx thickness. Specifically, the PBR of the sublingual arterial microvessels with diameters ranging from 5 to 25 μm was measured using Sidestream Dark Field imaging (Microscan, Glycocheck, Microvascular Health Solutions Inc., Salt Lake City, UT, USA), providing a rapid, non-invasive assessment of the endothelial glycocalyx thickness, as previously described [25]. This method, using specialized cameras, captures measurements from over 3000 vascular segments of sublingual microvessels in less than 3 min and has shown good reproducibility [25]. As such, the European Society of Cardiology Working Group on Peripheral Circulation has endorsed this technique as a reliable method for assessing endothelial integrity [25].

Moreover, carotid-femoral PWV, Aix, and central aortic pressures (central systolic and diastolic cSBP and cDBP) were measured using tonometry by Complior (Alam Medical, Vincennes, France). Normal values were defined as PWV < 10 m/s [27,28]. Aix was calculated as 100 × (P2 − P1)/PP, where P2 represents the late backward systolic wave, P1 the early forward systolic wave, and PP the pulse pressure. Aix reflects the pressure increase caused by the return of the reflected waves at the aorta [27].

### Statistical Analysis

For the statistical analysis, one eye per patient was included. If both eyes were affected by CRVO or BRVO, the right eye was selected in all cases, to avoid selection bias. Descriptive statistics were calculated to summarize patients’ characteristics. For normally distributed continuous variables, the mean ± standard deviation (SD) was reported, while for skewed distributions, the median and interquartile range (IQR) were used. Categorical variables were presented as relative frequencies and percentages. The Kolmogorov–Smirnov and the Shapiro–Wilk normality tests were applied for data distribution assessment. For each ophthalmological outcome (macular thicknesses, RNFL thicknesses, foveal avascular zone parameters, and vessel densities), we generated a saturated linear regression model with demographic and systemic vascular parameters serving as independent variables. The saturated models were reduced using a stepwise backwards elimination procedure, in which the variable yielding the highest *p* value over 0.05 was removed from the model, at each step. The procedure was terminated when all remaining variables had *p* values below 0.05. We report regression coefficients with the corresponding 95% confidence intervals (CIs). All analyses were performed with Statistical Package for Social Sciences (SPSS) software version 29.0 (IBM Corporation, Armonk, NY, USA). A *p* value < 0.05 was considered as statistically significant.

## 3. Results

Participants in the study were 28 consecutive patients with RVO, 12 patients (42.9%) with CRVO and 16 (57.1%) with BRVO. Table 1 shows the demographic and clinical characteristics of our study sample. The mean age of patients was 71.1 ± 10 years, while 14 out of 28 patients (50%) were male and 50% female. Regarding comorbidities, 82.1% of patients had hypertension, 50% diabetes mellitus and 53.6% dyslipidaemia.

The systemic vascular metrics are shown on Table 2. The median PBR5-25 was 2.21 μm (IQR: 2.12–2.34). The mean cSBP was 132.7 ± 16.1 mmHg, while the mean cDBP was 76.7 ± 8.6 mmHg. The PWV was measured to be 12.5 ± 3.2 m/s, and the median Aix was 14.90%.

The measurements for all retinal parameters on OCT and OCT-A are included in Table 3. The mean central subfield thickness was 317.5 ± 131.8 μm, while the median total macular thickness was 283 μm. The mean average RNFL thickness was 98.4 ± 18.3 μm. The mean macular vessel density (VD) was 39.74 ± 2.87%, while the mean FAZ area was 0.295 ± 0.135 mm^2^, with a FAZ circularity index of 0.534 ± 0.077. Regarding the optic disc OCT-A, the mean peripapillary VD was 44.19 ± 3.27%.

Table 4 shows the associations between the retinal imaging parameters and the systemic vascular metrics, using reduced multivariate models. A 1% increase in Aix is associated, on average, with a 2 μm decrease in superior macular thickness (*p* = 0.013). Female gender was associated, on average, with a 14 μm thicker average RNFL (*p* = 0.026) and with a 15 μm thicker temporal RNFL (*p* = 0.010). A 10 mmHg increase in cSBP is associated, on average, with a 5 μm increase in average RNFL thickness (*p* = 0.025), with a 6 μm increase in superior RNFL thickness (*p* = 0.029) and with a 4 μm increase in temporal RNFL (*p* = 0.019).

Regarding OCT-A parameters, an increase of 1 μm in the PBR5-25 was associated, on average, with an 9.6% increase in the inferior macular VD (*p* = 0.018). A 1 m/s increase in PWV was associated with a 0.6% reduction in inferior macular VD (*p* = 0.050). Hypertension was associated, on average, with a 0.21 mm^2^ decrease in FAZ area (*p* = 0.002). Smokers were associated, on average, with a 0.18 mm^2^ increase in FAZ area (*p* < 0.001). A 10 mmHg increase in cSBP was associated with a 0.03 mm^2^ increase in FAZ area (*p* = 0.033). A 1% increase in Aix was associated with a 0.005 mm^2^ increase in FAZ area (*p* = 0.008), while a 1 m/s increase in PWV was associated with a 0.017 mm^2^ decrease in FAZ area (*p* = 0.009).

Female gender was associated, on average, with a 3.7% higher peripapillary VD (*p* = 0.010), a 3.7% higher peripapillary superior VD (*p* = 0.019) and 5% higher peripapillary nasal VD (*p* < 0.001). A 1 μm increase in PBR5-25 was associated, on average, with a 4.4% decrease in superior peripapillary VD (*p* = 0.027). Diabetes was associated with 3.6% decrease in peripapillary nasal VD (*p* = 0.005). A 1 m/s increase in PWV was associated with a 0.5% increase in nasal peripapillary VD (*p* = 0.008). A 10 mmHg increase in cSBP was associated, on average, with a 0.1% increase in temporal peripapillary VD (*p* = 0.050).

## 4. Discussion

We report that, in patients with RVO, structural and microvascular retinal parameters were significantly associated with markers of endothelial damage and arterial stiffness, in complex patterns. In particular, increased PBR5-25 was associated with an increase in inferior macular VD, but a decrease in superior peripapillary VD. Increased Aix was associated with superior macular thinning and an increase in FAZ. Increased PWV was associated with a decrease in inferior macular VD, but an increase in nasal peripapillary VD and a decrease in FAZ. To our knowledge, this is the first study investigating the associations between the endothelial glycocalyx and retinal microvascular parameters in patients with RVO.

The endothelial glycocalyx is an important determinant of capillary blood flow, due to its impact on viscosity, which increases exponentially in vessels with narrow lumens [29,30]. The increase we report in inferior macular VD with increasing glycocalyx permeability could be attributed to local arteriolar or venular dilatation. Indeed, degradation of the glycocalyx in mouse models has shown acute increases in arteriolar and venular diameters with a concomitant decrease in venular shear rate [31]. Flow-mediated dilation of the arterioles could be related to endothelial dysfunction. Degradation of heparan sulfate chains would result in increased vasopermeability and impaired NO production [32]. Regarding venules, pressure-induced vasoexpansion has been suggested as a potential mechanism, but, in our case, occlusion of the larger downstream veins is a much more likely explanation [31]. The simultaneous decrease in superior peripapillary VD can be explained by the documented detrimental effect of RVO on the radial peripapillary capillaries, predisposing such patients to glaucomatous damage [33].

The association of arterial stiffness with increased odds for RVO, independently of age and arterial hypertension, has been previously reported [15,16,17,18,19]. Additionally, the magnitude of microvascular alterations in RVO patients has been shown to predict worse functional outcomes [20,21]. The decreasing superior macular thickness and increasing FAZ area with increasing Aix, as well as the decreasing inferior macular VD with increasing PWV that we report are in line with these findings, suggesting a potential involvement of vessel wall alterations in the pathogenesis of RVO. Counterintuitively, increased PWV was also associated with increased nasal peripapillary VD and decreased FAZ area. We postulate that preferential damage to the more vulnerable choroidal and optic nerve circulation could result in compensational redistribution of blood flow towards the more superficial layers.

Interestingly, in our population, higher SBP was associated with increased RNFL thickness and temporal peripapillary VD. Moreover, a diagnosis of hypertension and an increase in SBP had effects in opposite directions with regards to FAZ area. These observations are likely related to the documented U-shaped phenomenon, according to which not only high, but also low blood pressure could predispose to ganglion cell degeneration, especially in cases of aggressively treated arterial hypertension [34].

Regarding limitations, this study is cross-sectional; therefore, it is not possible to disentangle the temporal association between endothelial or vessel wall degradation and RVO development. In addition, likely due to its small sample size, the reported microvascular alterations were not consistently statistically significant in all retinal subsections examined. The small sample size also did not allow for the introduction of additional outcomes, while the multivariate analysis should be interpreted with caution based on the small sample size. Additionally, we did not perform electroretinography examination so as to identify potential markers for retinal function and microvascular alterations for the patients. Concerning the endothelial glycocalyx, there is currently no method to directly quantify its presence in the retinal microcirculation in vivo; thus, it was approximated using measurements from the sublingual microcirculation. Additionally, the limitations associated with quantitative OCT-A imaging, particularly its moderate repeatability, also apply to our study [35].

## 5. Conclusions

In conclusion, indicators of endothelial damage and impaired vessel wall compliance were linked to structural and microvascular changes in the retina in patients with RVO. These findings enhance our understanding of RVO pathophysiology, indicating that the degree of damage caused by RVO is not independent of the patient’s local and systemic vascular profile. Moreover, our findings could suggest that patients with RVO and retinal microvascular alterations are more likely to present with endothelial damage and arterial stiffness, resulting in potential cardiovascular problems. Further studies are necessary to establish whether these associations, aside from the pathophysiological perspective, are strong enough to predict clinical outcomes.

## Figures and Tables

**Table 1:** Demographic and clinical characteristics of the study population (*n* = 28).

Age (Mean ± SD, Years)	71.1 ± 10
Sex (*n*, %)	
Male	14 (50%)
Female	14 (50%)
Smoking (*n*, %)	11 (39.3%)
Hypertension (*n*, %)	23 (82.1%)
Diabetes mellitus (*n*, %)	14 (50.0%)
Dyslipidaemia (*n*, %)	15 (53.6%)

SD: standard deviation.

**Table 2:** Systemic vascular metrics in our study sample (*n* = 28).

**Glycocalyx PBR**	
5–25 μm [median (IQR)]	2.21 (2.12 to 2.34)
5–9 μm [median (IQR)]	1.30 (1.21 to 1.40)
10–19 μm [median (IQR)]	2.39 (2.18 to 2.50)
20–25 μm [median (IQR)]	2.92 (2.61 to 3.02)
**Vessel wall compliance**	
cSBP (mean ± SD, mmHg)	132.7 ± 16.1
cDBP (mean ± SD, mmHg)	76.7 ± 8.6
PP (mean ± SD, mmHg)	56.5 ± 16.5
Aix [median (IQR), %]	14.90 (11.76 to 28.08)
PWV (mean ± SD, m/s)	12.5 ± 3.2

Aix: augmentation index; cDBP: central diastolic blood pressure; cSBP: central systolic blood pressure; IQR: interquartile range; PBR: perfused boundary region; PP: pulse pressure; PWV: pulse wave velocity; SD: standard deviation.

**Table 3:** Retinal parameters in our study sample (*n* = 28).

**Macular Thickness (** **μm)**	
Central subfield thickness (mean ± SD)	317.5 ± 131.8
Parafoveal superior (mean ± SD)	280.7 ± 49.2
Parafoveal inferior (mean ± SD)	299.4 ± 92.0
Parafoveal nasal (mean ± SD)	306.0 ± 53.7
Parafoveal temporal (mean ± SD)	286.6 ± 73.4
Perifoveal superior (mean ± SD)	321.2 ± 67.1
Perifoveal inferior (mean ± SD)	345.5 ± 116.9
Perifoveal nasal (mean ± SD)	343.8 ± 82.8
Perifoveal temporal (mean ± SD)	339.0 ± 104.9
Total [median (IQR)]	283 (263 to 357)
Superior [median (IQR)] ^×^	286 (262 to 304)
Inferior [median (IQR)] ^×^	296 (261 to 344)
Nasal [median (IQR)] ^×^	303 (274 to 341)
Temporal [median (IQR)] ^×^	281 (259 to 317)
**RNFL thickness** **(μ** **m)**	
Average (mean ± SD)	98.4 ± 18.3
Superior (mean ± SD)	117.3 ± 23.3
Inferior (mean ± SD)	122.4 ± 28.5
Nasal (mean ± SD)	80.3 ± 21.8
Temporal (mean ± SD)	74.0 ± 17.4
**Macular OCT-A**	
mVD average (%, mean ± SD)	39.74 ± 2.87
Superior (%, mean ± SD)	38.42 ± 4.60
Inferior (%, mean ± SD)	40.14 ± 5.23
Nasal (%, mean ± SD)	41.50 ± 5.21
Temporal (%, mean ± SD)	38.90 ± 6.39
FAZ area (mm^2^, mean ± SD)	0.295 ± 0.135
FAZ perimeter (mm, mean ± SD)	2.564 ± 0.623
FAZ circularity index (mean ± SD)	0.534 ± 0.077
**Peripapillary OCT-A**	
pVD average (%, mean ± SD)	44.19 ± 3.27
Superior (%, mean ± SD)	46.05 ± 5.29
Inferior (%, mean ± SD)	46.58 ± 4.14
Nasal (%, mean ± SD)	40.95 ± 4.73
Temporal (%, mean ± SD)	43.19 ± 4.46

^×^ Average of parafoveal and perifoveal measurements. FAZ: foveal avascular zone; IQR: interquartile range; mVD: macular vessel density; OCT-A: optical coherence tomography angiography; pVD: peripapillary vessel density; RNFL: retinal nerve fiber layer; SD: standard deviation.

**Table 4:** Associations between retinal parameters and systemic vascular metrics: reduced multivariable models.

**Macular Thickness**		
	**Beta (95% CI)**	***p* Value**
**Total, μm**		
NA	NA	NA
**Central subfield thickness, μm**		
NA	NA	NA
**Superior, μm**		
Aix, per 1% increase	−2 (−3 to 0)	0.013
**Inferior, μm**		
NA	NA	NA
**Nasal, μm**		
NA	NA	NA
**Temporal, μm**		
NA	NA	NA
**RNFL thickness**		
**Total, μm**		
Gender (Ref: female)	−14 (−26 to −2)	0.026
cSBP, per 10 mmHg increase	5 (1 to 9)	0.025
**Superior, μm**		
cSBP, per 10 mmHg increase	6 (1 to 11)	0.029
**Inferior, μm**		
NA	NA	NA
**Nasal, μm**		
NA	NA	NA
**Temporal, μm**		
Gender (Ref: female)	−15 (−27 to −4)	0.010
cSBP, per 10 mmHg increase	4 (1 to 8)	0.019
**Macular OCTA**		
**mVD total, %**		
NA	NA	NA
**Superior mVD, %**		
NA	NA	NA
**Inferior mVD, %**		
Glycocalyx total PBR, per μm increase	9.6 (1.8 to 17.4)	0.018
PWV, per m/s increase	−0.6 (−1.1 to 0)	0.050
**Nasal mVD, %**		
NA	NA	NA
**Temporal mVD, %**		
NA	NA	NA
**FAZ area, mm^2^**		
Hypertension (Ref: normotension)	−0.21 (−0.34 to −0.09)	0.002
Smoking (Ref: no smoking)	0.18 (0.08 to 0.27)	<0.001
cSBP, per 10 mmHg increase	0.03 (0 tο 0.05)	0.033
Aix, per 1% increase	0.005 (0.001 to 0.009)	0.008
PWV, per m/s increase	−0.017 (−0.029 to −0.005)	0.009
**Peripapillary OCTA**		
**pVD total, %**		
Gender (Ref: female)	−3.7 (−5.8 to −1.6)	0.010
**Superior pVD, %**		
Glycocalyx total PBR, per μm increase	−4.4 (−8.1 to −0.8)	0.027
Gender (Ref: female)	−3.7 (−5.8 to −1.6)	0.019
**Inferior pVD, %**		
NA	NA	NA
**Nasal pVD, %**		
Gender (Ref: female)	−5.0 (−7.4 to −2.5)	<0.001
Diabetes mellitus (Ref: no diabetes)	3.6 (1.2 to 6.0)	0.005
PWV, per m/s increase	0.5 (0.2 to 0.9)	0.008
**Temporal pVD, %**		
cSBP, per 10 mmHg increase	0.1 (0.0 tο 0.2)	0.050

Aix: augmentation index; CI: confidence intervals; cSBP: central systolic blood pressure; FAZ: foveal avascular zone; mVD: macular vessel density; NA: not applicable—all variables removed from model; OCTA: optical coherence tomography angiography; PBR: perfused boundary region; pVD: peripapillary vessel density; PWV: pulse wave velocity; RNFL, retinal nerve fiber layer.

## Data Availability

Data are unavailable due to privacy restrictions.
